# Experimental mouse models for translational human cancer research

**DOI:** 10.3389/fimmu.2023.1095388

**Published:** 2023-03-10

**Authors:** Yinxi Zhou, Jinghua Xia, Shuonan Xu, Tao She, Yanning Zhang, Ying Sun, Miaomiao Wen, Tao Jiang, Yanlu Xiong, Jie Lei

**Affiliations:** Department of Thoracic Surgery, The Second Affiliated Hospital, Air Force Medicel University, Xi’an, China

**Keywords:** tumor, immune microenvironment, animal model, spontaneity, induced, transgene, PDX, immunodeficiency

## Abstract

The development and growth of tumors remains an important and ongoing threat to human life around the world. While advanced therapeutic strategies such as immune checkpoint therapy and CAR-T have achieved astonishing progress in the treatment of both solid and hematological malignancies, the malignant initiation and progression of cancer remains a controversial issue, and further research is urgently required. The experimental animal model not only has great advantages in simulating the occurrence, development, and malignant transformation mechanisms of tumors, but also can be used to evaluate the therapeutic effects of a diverse array of clinical interventions, gradually becoming an indispensable method for cancer research. In this paper, we have reviewed recent research progress in relation to mouse and rat models, focusing on spontaneous, induced, transgenic, and transplantable tumor models, to help guide the future study of malignant mechanisms and tumor prevention.

## 1 Introduction

According to the World Health Organization, there were 19.3 million new cancer cases worldwide in 2020, resulting in nearly 10 million related deaths (1). Furthermore, with the acceleration in population growth and social aging, the global cancer burden will continue to increase. It is estimated that by 2040, the number of newly diagnosed cancer patients will reach 28.4 million per year, an increase of 47% when compared with 2020 (2). Recently, new therapeutic strategies, such as immunotherapies and targeted therapies, have significantly improved the survival rates of tumor patients when compared with traditional therapies; however, there are fewer sensitive and beneficial people, and the problem of drug resistance is almost inevitable (3). Therefore, in-depth exploration of the occurrence and development mechanisms of cancer and the improvement of diagnosis and treatment strategies are of critical global importance, and consequently, this is a major focus of academic research.

Animal tumor models are valuable for experimental analysis as the animal tumors can have similar characteristics to human tumors. We can study the occurrence and development of tumors by observing animal tumor models, and study the effect of genes on tumorigenesis and development by knocking out or knocking in a certain gene, and we can also establish PDX models for drug screening and preclinical trials. Furthermore, this experimental strategy is also advantageous as these experiments are low cost, the animals have short life cycles, and it can help to avoid human experiments. Thus, the use of animal models is extremely valuable for tumor research (4). At present, based on the causes of tumor formation, we divide the animal model into four categories: spontaneous, induced, transgenic, and transplant. Among them, the spontaneous tumor model, the induced tumor model, and the transgenic model belong to the primary tumor of animals, and the transplantable tumor models include allogeneic transplantation and xenogeneic transplantation; the most used transplantable tumor model is human tumor xenografts in immunodeficient mice. Compared with non-mammals, mammals have a higher degree of similarity to humans, especially mice and rats, and the short reproduction cycle of mice greatly improves the efficiency of these experiments. The mouse genome sequencing project was completed in 2002, and it revealed that 99% of human genes exist in mice; gene homology is as high as 78.5% (5). Consequently, mice are the most commonly used animals in tumor research. This paper introduces the research progress that has been made with various human tumor models in mice and rats, and analyzes their development processes, advantages and disadvantages, applications, and future development prospects.

## 2 Animal models for spontaneous tumors

**Figure 1:**
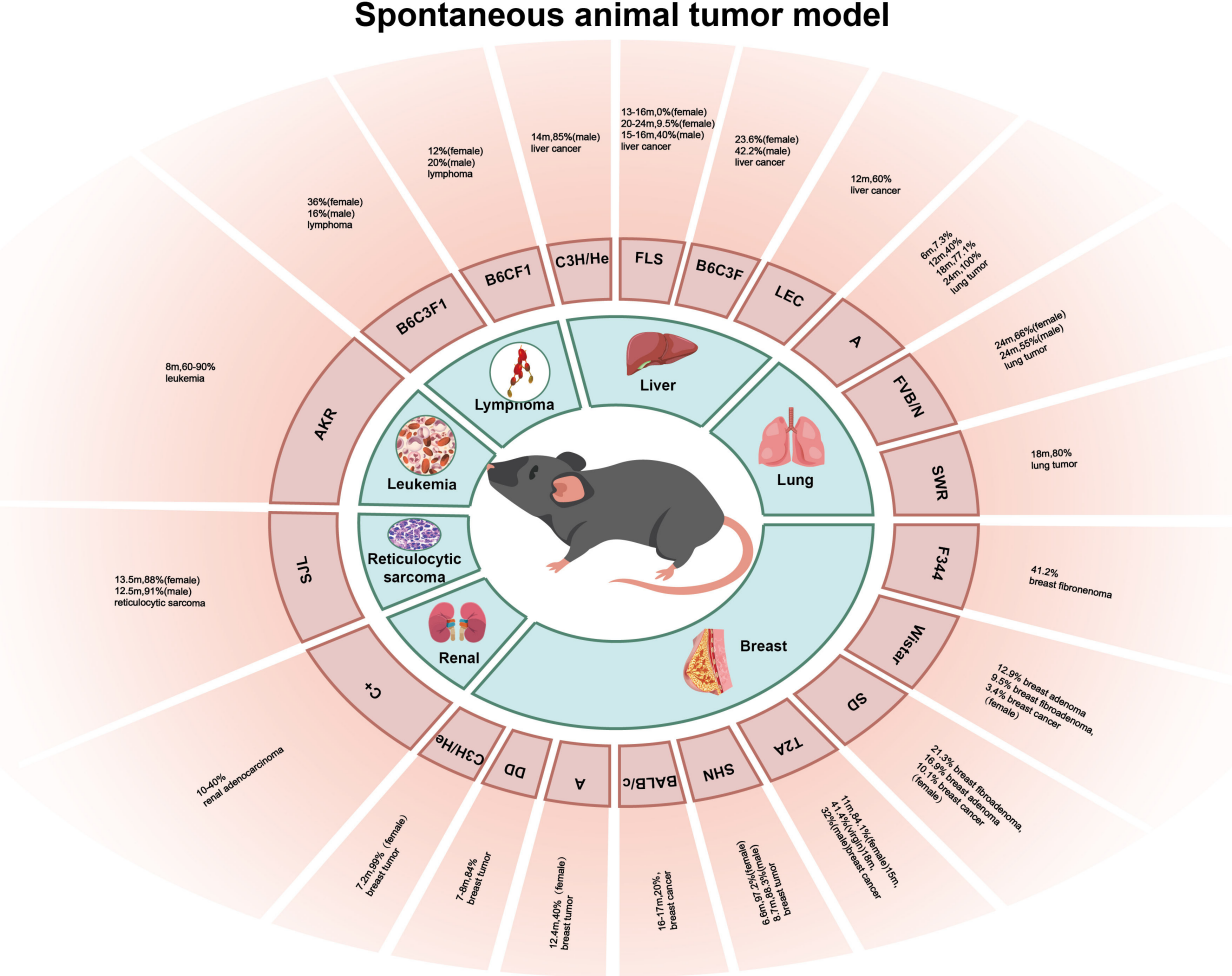
The incidence of various spontaneous tumors in different strains of mice (rat).

Spontaneous animal tumor models are utilized to investigate tumors that occur naturally in experimental animals without any conscious artificial intervention, and this occurrence type and incidence vary greatly with different species and strains of experimental animal (**Figure 1**). The advantage of the spontaneous animal tumor model is that the process of tumor formation is similar to that of human tumors, and the long period of tumor occurrence and development can be treated comprehensively with the intervention of many factors, and the role of genetic factors in tumorigenesis can be observed. However, the disadvantage of the spontaneous animal tumor model is that the experimental cycle is long, many experimental animals are required, and the cost is high; in addition, the spontaneous tumors of animals are often heterogeneous and there are individual growth differences, making it difficult to obtain a large number of tumor-bearing animals with uniform growth at the same time.

### 2.1 Breast tumors

The incidence and mortality of breast cancer worldwide are increasing and have surpassed lung cancer in 2020 to become the most predominant cancer in the world (1). Mice have a high incidence of breast cancer (6). The occurrence of breast tumors in mice is affected by many factors, including factors such as viruses, hormones, heredity, and feed (7). Mouse mammary tumor virus (MMTV) is the most common cause of breast cancer, which causes breast cancer and lymphoma in mice mainly by insertion mutations and clone amplification in the genome; therefore, mice carrying MMTV have a higher incidence of breast tumors (8). C3H/He mice were inbred by Strong after crossing Bagg albino female mice with a high incidence of the breast tumor strain DBA male mice in 1920. C3H/He female mice carried MMTV, and the average incidence of spontaneous breast tumors reached 99% at an average of 7.2 months, but the average age of tumorigenesis in the virgin mice was approximately 2 months older than that in postpartum mice (9). DD mice also carry MMTV, and the incidence of breast tumors is 84% at the age of 7–8 months (9). BALB/c and other mouse strains do not carry MMTV, and the incidence of breast tumors is low; in 1932, the 26th generation of mice bred by MacDowell from Bagg albino mice through inbred lines was named BALB/c mice, and the incidence of breast cancer in the BALB/c mice was only 20% at 16–17 months (9), and the incidence of breast tumors in C57BR, C57BL, AKR, and other mice without MMTV was lower (10). However, some strains of mice without MMTV had a high incidence of breast tumors, such as the A mice, SHN mice, and TA2 mice; A mice were inbred by Strong after inbreeding with the offspring of the local albino mice and Bagg albino mice in 1921, which is a highly spontaneous breast tumor strain, but the A mice do not carry MMTV, and Strain A female mice had an average breast tumor incidence of 40% at the age of 12.4 months (9). SHN mice were bred by Nagasawa et al. on the basis of the Swiss albino mice, and the incidence of breast tumors in female SHN mice was 97.2% at an average age of 6.6 months, and 88.3% in virgin female SHN mice at 8.7 months (10). In addition, the inbred strain TA2 mice bred by Sun et al. also had a high incidence of spontaneous breast cancer, and pathological analysis showed that the breast cancer cells of the TA2 mice were triple-negative. The incidence of spontaneous breast cancer reached 84.1% after 11 months in the TA2 mice, 41.4% in the virgin TA2 mice at 15 months, and 32% in the male TA2 mice at 18 months (11). Wistar, SD, F344, and other rat strains also had a high incidence of breast cancer; Wistar rats were bred by the Wistar Institute of the United States in 1907, and SD rats were bred by Wistar rats in 1925. The incidences of breast fibroadenoma, breast adenoma, and breast cancer in female SD rats were 21.3%, 16.9%, and 10.1%, respectively, while those in female Wistar rats were 12.9%, 9.5%, and 3.4%, respectively (12). F344 rats were inbred and established by Dunning in subline 344 of the Fischer strain, and the incidence of breast fibroadenoma in the F344 rats was 41.2% (13).

### 2.2 Lung tumors

Lung cancer is one of the most common cancers and the leading cause of cancer-related death worldwide, with an estimated 2 million new cases and 1.76 million deaths annually (14). The histopathological subtypes of lung cancer include non-small cell lung cancer (NSCLC) and small cell lung cancer (SCLC), which can be further divided into adenocarcinoma and squamous cell carcinoma (SCC) (15). The incidence of spontaneous lung tumors in mice is high, and the pathological types are mainly adenoma and adenocarcinoma, depending on the mouse strain. A mice and SWR mice have a higher incidence of lung tumors, followed by BALB/C mice, CBA mice, and C3H mice, which have a lower incidence of lung tumors, and the lowest incidence of lung tumors was observed in DBA and C57BL/6 mice (16). A mice are a high spontaneous lung tumor strain, and lung tumors can be detected at 3–4 weeks of age. The incidence of lung tumor was 7.3% (6/83) at 6 months of age, 40.0% (71/178) at 12 months of age, 77.1% (105/136) at 18 months, and almost 100% at 24 months (17). SWR mice were bred by Lynch et al. in 1926 using Swedish mice for inbreeding. The spontaneous rate of lung tumors in SWR mice over 18 months was 80%, and the incidence of lung tumors in mice whose parents had spontaneous lung tumors was higher than that in mice whose parents had lung tumors alone, while the incidence of lung tumors in mice whose parents had no lung tumors was lower (18), suggesting a role for genetic factors in tumorigenesis. Although the incidence of lung tumors in SWR mice is high, the long culture period limits the use of this strain in lung tumor research. FVB/N mice were established in the 1970s, and they have a high spontaneous lung tumor rate. The mean incidence of lung tumors in male and female mice at 24 months was 55% and 66%, respectively, and the fertilized egg of FVB/N mice has a large and significant pronucleus and is easy to be microinjected with DNA. Consequently, FVB/N mice have been widely used as animal tumor models (19).

### 2.3 Liver tumors

Primary liver cancer is the fourth leading cause of cancer death worldwide, and its incidence is increasing every year. Histologically, it can be divided into hepatocellular carcinoma (HCC) and intrahepatic cholangiocarcinoma (20). Spontaneous liver tumors in mice often originate from hepatocytes, cholangiocarcinoma is rare, and sarcomas are even rarer. The incidence of liver tumors is different in different strains of mice, but the incidence in male mice is usually higher than that in female mice (21). Male C3H/He mice are a highly spontaneous liver tumor strain, and Heston et al. found that the incidence of liver cancer in 14-month-old male C3H/He mice could reach 85% (9). In Japanese FLS mice, inbred mice with non-obese spontaneous fatty liver developed a fatty liver shortly after being fed a normal diet, but were not visibly obese; multiple white protruding nodules appeared in the liver of mice over 12 months of age, and were histologically diagnosed as hepatocytic adenoma and HCC. The incidence of HCC in male mice was 40% at an average of 15–16 months, and that in female Japanese FLS mice was 0% (0/36) at 13–16 months and 9.5% (4/42) at 20–24 months (22). B6C3F1 mice are the first generation of female C57BL/6 and male C3H hybridizations, and the incidence of liver tumor was 42.2% in male B6C3F1 mice and 23.6% in female B6C3F1 mice (13). LEC rats are inbred mutant rats established by Joseph A. Long and Herbert M.E. Vans of the University of California, USA. Approximately 40% of LEC rats develop acute posthepatitic death 3–4 months after birth and approximately 60% of rats experience chronic hepatitis and develop HCC a year later (23, 24). Some studies have pointed out that the cause of spontaneous hepatitis and HCC in LEC rats is the excessive accumulation of copper in the liver, which produces a large amount of ROS hydroxyl radicals, followed by oxidative stress, which is similar to the development of HCC in humans. Therefore, LEC rats have been widely used in HCC models (25).

### 2.4 Other tumors

In 1958, Claude et al. described an animal model of renal adenocarcinoma in C+ mice. The incidence of renal adenocarcinoma in C+ mice was between 10% and 40%, and the incidence of tumors increased with age (26). The AKR mouse is a new strain established by further inbreeding of AK mice by the Rockefeller Institute in 1936; AKR mice are born with carcinogenic RNA virus, the incidence of leukemia after 8 months is approximately 60%–90%, and that of 18-month-old mice can be as high as 90% (27). The incidence of reticulocytic sarcoma in the SJL mice was high, and the incidence of reticulocytic sarcoma in 13.5-month-old female and 12.5-month-old male mice was 88% and 91%, respectively (28). Dragani et al. established hybrid mice (C57BL/6J × C3Hf) F1 (B6C3F1) and (C57BL/6J × BALB/c) F1 (B6CF1); the incidence of lymphoma in male B6C3F1 and B6CF1 mice was 16% and 20%, respectively, and that in female B6C3F1 and B6CF1 mice was 36% and 12%, respectively (21).

## 3 Animal models of induced tumor

**Figure 2:**
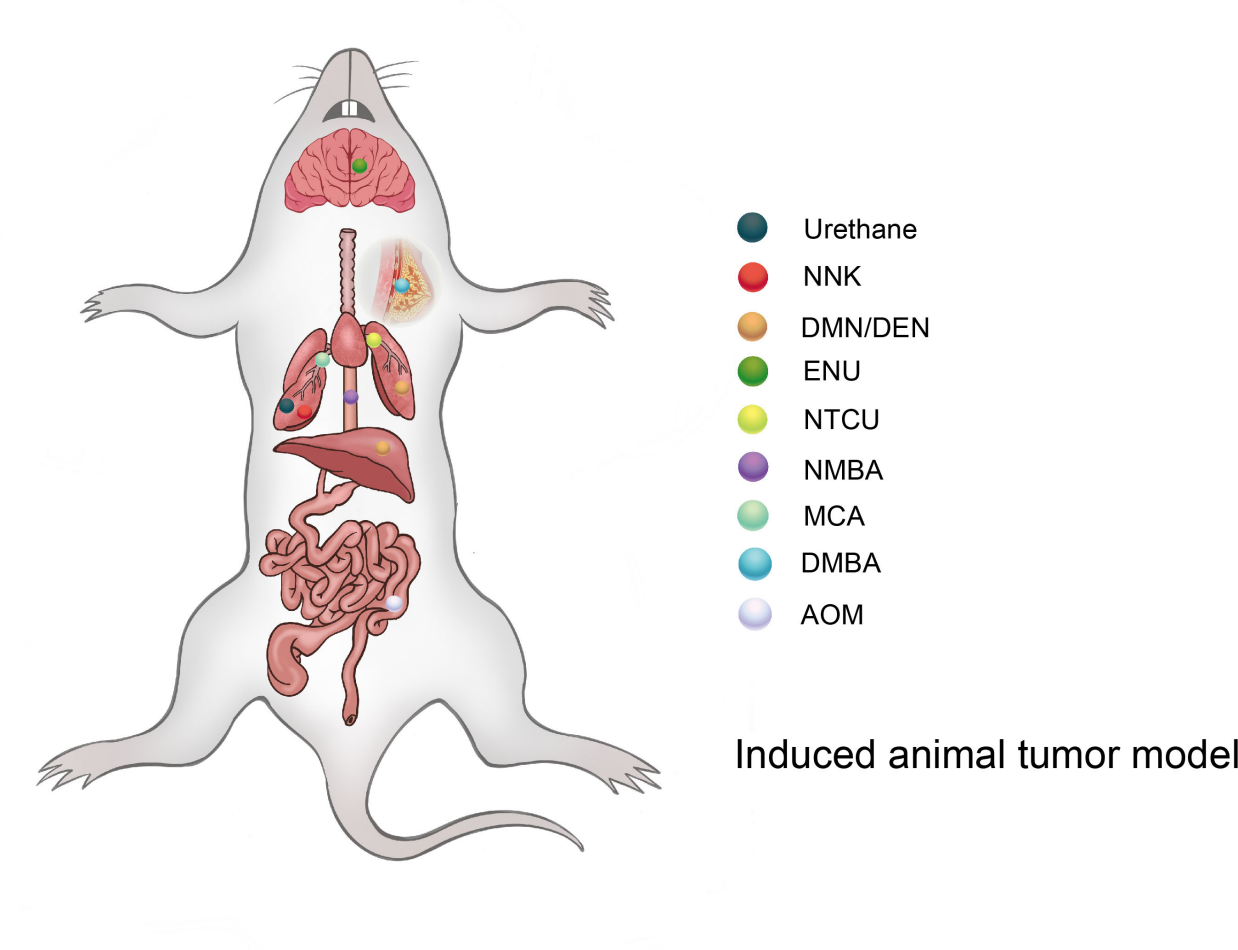
Different types of compounds can induce tumors in different organs of mice (rat).

The induced tumor model refers to the use of exogenous carcinogens to cause changes in the cellular genetic characteristics, resulting in the abnormal growth of active cells and the formation of tumors (**Figure 2**). Induction methods include physical, chemical, and biological methods, of which the chemical methods are the most extensive and effective for the induction (**Table 1**), and have the advantages of easy application, short experimental time, and high reproducibility (45). However, the disadvantage is a high animal mortality rate, and the time, location, and number of lesions are not uniform among individuals.

**Table 1:** Construction method of animal tumor model induced by chemical drugs.

Chemical	Species	Injection	Dose	Type of tumor	Incidence	Reference
Urethan	A mice	i.p.	1,000 mg/kg, single injection	Lung tumor	6 months: 75%	(29)
	BALB/c mice	i.p.	600 mg/kg, three times every 2 days	Lung tumor	20 weeks: 70%	(30)
	Swiss mice (newborn)	Breast-feeding	Parental female mice were administered 30 mg in 1, 3, and 5 days after delivery	Lung tumor	3 months: 52%; 7 months: 78%	(31)
	Swiss mice (newborn)	i.h.	2 mg, single injection	Lung tumor	3 months: 100%	(31)
	C57BL/6J mice	i.p.	1,000 mg/kg, once a week for 10 weeks	Lung tumor	30 weeks: 100%	(32)
NNK	A/J mice	i.p.	100 mg/kg, single injection	Lung adenomas	34–42 weeks: 50%	(33)
	A/J mice	i.p.	3 μmol weekly for 8 weeks	Lung adenomas	26 weeks: 100%	(34)
	Swiss mice (newborn)	i.p.	50 mg/kg, on postnatal days 1, 4, 7, 10, and 14	Lung tumor	13–15 months: 57% (male), 37% (female)	(35)
DEN	FVB/N mice (15-day-old)	i.p.	15 μg/g, single injection	Lung cancer (papillary cancer)	12 months: 72%	(36)
	C57BL/6 mice (male)	i.g.	0.014% DEN for 6 days a week	Hepatocellular carcinoma	11–15 weeks: 100%	(37)
DEN+ CCl4	B6C3F1/J mice(male)	i.p. (CCl4) i.v. (DEN)	1 mg/kg DEN, 40.2 ml/kg CCL4 every week from week 8 to 14	Liver adenoma/liver cancer	22 weeks: 100% (liver adenoma), 50% (liver cancer)	(38)
ENU	SD rats (offspring)	i.v. (pregnant rats)	50 mg/kg, on day 20 of their pregnancy	Nervous system tumor	1 year: 100%	(39)
NTCU	SWR/J mice	Skin smear	25-μl drop of 0.04 M, twice a week for 8 months	Lung squamous cell carcinoma	8 months: 100%	(40)
	NIH Swiss mice	Skin smear	25-μl drop of 0.04 M, twice a week for 8 months	Lung squamous cell carcinoma	8 months: 83%	(40)
	A/J mice	Skin smear	25-μl drop of 0.04 M, twice a week for 8 months	Lung squamous cell carcinoma	8 months: 75%	(40)
	FVB/J mice	Skin smear	25-μl drop of 0.04 M, twice a week for 8 months	Lung squamous cell carcinoma	8 months: 44%	(40)
	BALB/cJ mice	Skin smear	25-μl drop of 0.04 M, twice a week for 8 months	Lung squamous cell carcinoma	8 months: 38%	(40)
DMBA	SD rats	i.g.	80 mg/kg, gavaged once	Breast cancer	12 weeks: 100%	(41)
NMBA	SD rats	i.h.	0.5 mg/kg, three times a week for 5 weeks or once a week for 15 weeks	Esophageal tumors	20 weeks: 100%	(42)
MCA	BC3Fl mice	Tracheal drip	0.5 mg, 6 times a week	Respiratory tract squamous cell carcinoma	10–28 weeks: 86%	(43)
	DBA/2 mice	Tracheal drip	0.5 mg, 6 times a week	Respiratory tract squamous cell carcinoma	7 months: 6%	(43)
AOM	A/J or FVB/N mice	i.p.	10 mg/kg, once a week for 6 weeks	Colon tumors	30 weeks: 80%–100%	(44)

### 3.1 Urethan

Urethan was originally used as a herbicide and was later found to inhibit cell division and, thus, utilized as a chemotherapeutic drug for leukemia (46). Urethan is structurally one of the simplest carcinogens; it is soluble in both water and lipids and was the first water-soluble carcinogen to be discovered (47). The biological effects of urethan depend on the direct inhibition of nucleic acid synthesis, especially of the pyrimidine bases, and an apparent antagonism between urea and pyrimidines also exists (47). In the 1950s, a study on a model of lung tumors induced by urethan as a carcinogen found that 100% of highly sensitive mice could induce lung tumors (48). Mice are highly sensitive to urethan, especially newborn mice, and studies have shown that the rate of clearance of the urethan in newborn mice is 1/10 of that in adult mice, and 0.5 mg/g urethan can be metabolized in adult mice within 8 h, while the same dose takes 72 h in newborn mice (49). Strain A mice have highly spontaneous lung tumor development; while >75% of mice can develop lung tumors at 18 months, 75% of A mice can develop lung tumors at 6 months after a single injection of 1,000 mg/kg urethan (29). For BALB/c mice, Koohdani et al. injected 600 mg/kg intraperitoneally with urea three times every 2 days and the incidence of lung tumors in the BALB/c mice reached 70% at 20 weeks (7/10) (30). In 1962, De Benedictis et al. induced lung tumors in Swiss mice, and the parental female mice were administered 30 mg of urea 1, 3, and 5 days after delivery; the results showed that the incidence of lung tumors in the offspring was 52% and 78% at 3 months and 7 months, respectively, and another group of offspring received a single subcutaneous injection of 2 mg in the interscapular area within 24 h after birth, and the incidence of lung tumor was 100% in 3 months (31). However, the C57BL/6 mice commonly used in genetic engineering have high resistance to lung tumors induced by urethan, and consequently, multiple injections of urethan are required to overcome their genetic resistance and induce lung cancer. C57BL/6J mice were intraperitoneally injected with 1,000 mg/kg once a week for 10 weeks, and the results showed that the incidence of lung tumors in C57BL/6J mice was close to 100% at 30 weeks (32).

### 3.2 NNK

NNK, also known as (methylnitrosamine)-1-(3-pyridine)-1-butanone, is one of the main chemical carcinogens in cigarette smoke (50), and it can effectively induce lung cancer in mice, rats, and hamsters (51). The α-hydroxylation of NNK catalyzed by a cytochrome 450 scan easily binds to DNA and forms DNA adducts, causing gene mutations that lead to tumor development (33, 52). Belinsky et al. showed that in 6-week-old A/J mice, a single intraperitoneal injection of 100 mg/kg NNK resulted in the development of hyperplasia along the alveolar septum after 14 weeks, and the frequency of lung adenomas in A/J mice at 34–42 weeks resulted in a significant increase to 50% (53). In another independent study, 7-week-old female A/J mice were given intraperitoneal injections of NNK 3 μmol weekly for 8 weeks, and 100% of the mice developed lung adenomas after 26 weeks (34). NNK can also induce lung cancer in offspring, as Anderson et al. gave 100 mg/kg NNK doses to female A/J mice on days 14, 16, and 18 of their pregnancies, and it was found that lung tumors occurred in 12 of the 66 offspring and 13 of the 14 female mice (54). The effects of NNK on young mice were also studied in Swiss mice. Mice were given 50 mg/kg NNK i.p. on postnatal days 1, 4, 7, 10, and 14, and the incidence of lung tumors was 57% in male mice and 37% in female mice at 13–15 months (35).

### 3.3 DMN/DEN

Dimethylnitrosamines (DMN) and diethylnitrosamines (DEN) are nitrosamines, which can induce various tumors in mice and rats, but predominantly liver and lung tumors. Nitrosamines are activated by CYP450 enzymes *in vivo* and converted into a strong alkylating agent, forming adducts in the DNA (active chemicals and cellular macromolecules form stable complexes through covalent bonds), resulting in carcinogenicity (55). Kohda et al. confirmed the mutagenicity of nitrosamines by treating Chinese hamster V79 cells with nitrosamines (56). After DEN exposure, A/J mice developed lung adenocarcinoma, 82% of which possessed KRAS mutations (57). FVB/N mice also have a high incidence of lung cancer, as Zsolt injected 15 μg/g DEN intraperitoneally into 15-day-old FVB/N mice, and the incidence of lung cancer (papillary cancer) was 72% (28/39) after 12 months. Interestingly, there were no mutations in the Kras and EGFR genes (36). In a study by Chen et al., C57BL/6 male mice were intragastrically administered 0.014% DEN for 6 days a week and given normal drinking water on the 7th day for 15 weeks, and 100% of these mice developed fibrosis (3–6 weeks), cirrhosis (7–10 weeks), and HCC (11–15 weeks) at 3–15 weeks (37). The incidence of liver tumors caused by the combination of DEN and CCl4 was significantly higher than that of DEN or CCl4 alone. Uehara et al. used 14-day-old B6C3F1/J male mice and administered 1 mg/kg DEN intravenously, and 40.2 ml/kg CCL4 was injected intraperitoneally every week from weeks 8 to 14. The incidence of liver adenoma and liver cancer was 40% and 20% at 17 weeks, and the incidence of liver adenoma and liver cancer was 100% and 50% at 22 weeks (38). In 1974, Cardesa et al. divided 8-week-old Swiss mice into two groups: subcutaneous injection of DMN or DEN 8 mg/kg; the results showed that the incidence of tumors in mice was 79% (31/39) and 87% (34/39), and the incidence of lung tumors was 67% and 61%, respectively. Lung tumors in the two groups were mainly adenocarcinoma, adenoma, and atypical adenomatoid hyperplasia (58).

### 3.4 ENU

Acetylnitrosourea (ENU) is a strong mutagenic agent that can cause rapid oncogenic genetic mutations in mice (59). Raju et al. reported that SD rats were administered a single intravenous injection of ENU (50 mg/kg) on day 20 of their pregnancy, and almost 100% of the offspring had central and peripheral nervous system tumors at 1 year of age (39).

### 3.5 NTCU

N-nitroso-tris-chloroethylurea (NTCU) is a nitrosoalkylurea compound, which has been shown to induce lung SCC in mice. Wang et al. treated eight different strains of female mice by smearing NTCU on the skin to establish a model. In their study, the back skin of 7-week-old mice was scraped, and they were injected with NTCU 25-μl drop of 0.04 M, twice a week, with a 3-day interval; 8 months later, five strains of the mice had successfully induced lung SCC *in situ* or lung SCC [the induction rates were as follows: SWR/J, 100% (3/3); NIH Swiss, 83% (10/12); A/J, 75% (6/8); FVB/J, 44% (4/9); and BALB/cJ, 38% (3/8)]. The other strains (AKR/J, 129/svJ, and C57BL/6J) failed to develop the carcinomas, and histological and pathological analysis showed that mouse SCC induced by NTCU had the same pathological process as human SCC, which is “normal-proliferative-metaplastic-abnormal-SCC” (40, 60).

### 3.6 DMBA

Dimethylbenzanthracene (DMBA) is frequently used as a model for polycyclic aromatic hydrocarbon (PAH)-induced mammary tumorigenesis because of its potent carcinogenic and immunosuppressive activities (61). Female SD rats at the age of 7 weeks were diluted with 80 mg/kg DMBA in 0.5 ml of corn oil and gavaged once, and after 12 weeks, animal models of breast cancer lesions could be established in all rats (41).

### 3.7 NMBA

Methyl benzylnitrosamine (NMBA) is also an important carcinogenic compound that is classified as a nitrosamine. NMBA is currently the most effective inducer of rat esophageal tumors, as they can be induced in 15 weeks or less (62). SD rats were given 0.5 mg/kg NMBA three times a week for 5 weeks or once a week for 15 weeks, and the esophageal tumor incidence was 100% at 20 weeks (42).

### 3.8 MCA

Methylcholanthracene (MCA) is a potent carcinogen that is often used to induce the transformation of cultured cells, and it was found that repeated intratracheal injection of MCA into BC3Fl [(C57BL×C3H)F1] and DBA/2 mice could induce respiratory tract SCC, and the induced SCC had obvious infiltration and metastasis (43). BC3F1 mice were injected with 0.5 mg of MCA six times a week, and the incidence of respiratory tract SCC was 86% at 10–28 weeks. In contrast, DBA/2 mice that received intratracheal injections of 0.5 mg of MCA four times a week resulted in 6% incidence of SCC of the respiratory tract at 7 months (43).

### 3.9 AOM

Azomethane (AOM) is a chemical reagent that can promote base mismatch and cause cancer through the alkylation of DNA, which is often used in colonic carcinogenicity (63); 10 mg/kg AOM was injected intraperitoneally into A/J or FVB/N mice once a week for 6 weeks, and resulted in 80%–100% of mice having spontaneous colon tumors within 30 weeks (44).

### 3.10 Fattening diet

Nonalcoholic fatty liver disease (NAFLD) is a condition characterized by the excessive accumulation of fat in the liver without chronic alcohol intake; it is estimated that the global prevalence rate is approximately 24% (64). NAFLD and nonalcoholic steatohepatitis (NASH) are the liver manifestations of metabolic syndrome, and some patients with NAFLD develop into NASH with associated inflammation and fibrosis, which can progress to HCC (64). Asgharpour et al. successively established a model by following a fattening diet (high fructose-glucose solution and high-fat, high-carbohydrate diet—42% calorie fat and 0.1% cholesterol) in 8- to 12-week-old male mice; 89% of the mice developed HCC at 32–52 weeks, and each mouse had three or more tumor foci (65).

### 3.11 Ionizing radiation

Moderate to high doses of radiation are well-established causes of cancer (66), and ionizing radiation such as x-rays, α-rays, β-rays, and γ-rays can break through genetic material and cause DNA fracture damage and gene mutations (67). Some studies have shown that irradiated mice may develop a series of malignant tumors, including sarcomas, and single high-dose radiation significantly increases the incidence of tumors when compared with fractionated radiation (68). Edmondson et al. locally irradiated the right hindlimb of C3Hf/Kam and C57BL/6J mice with a high dose (10–70 Gy) or a fractionated dose (40–80 Gy, 2 Gy per day, five times a week, for 4–8 weeks), and after 800 days, 210 tumors were induced in 788 mice. The observed tumors were primarily sarcoma (*n* = 201), and the occurrence frequency of cancer was low (*n* = 9). The incidence of tumor after single irradiation was 36.1%, and that of graded irradiation was only16.4% (68).

## 4 Transgenic models

In recent years, increasing evidence has shown that gene mutation is an important cause of tumorigenesis, and targeted therapies for driving genes have achieved good results in tumor patients with a specific genetic background, but the problem of drug resistance still restricts the further benefits (3). Understanding the mechanism of driving mutations in tumorigenesis and development is thus crucial. Due to the high similarity between mouse and human protein coding genes, transgenic mice have been used to study the effects of gene mutations on tumorigenesis and development since the mid-1980s (5) (**Table 2**). The advantages of transgenic animal tumor models are that they have great advantages in studying tumorigenesis mechanisms and tumor immune escape. However, the establishment process of transgenic animal models is long, the feeding costs are high, and it is difficult to obtain a large number of experimental animals, which hinders rapid and high-throughput research (76).

**Table 2:** The type of tumor caused by gene mutation.

Gene mutations	Type	Tumor	Common mutation sites	References
KRAS	Proto-oncogene	Pancreatic cancer (70%–90%), colon cancer (50%), lung cancer (25%–50%)	G12D, G12V, G12C, G13D, AMP, G12R	(69, 70)
TP53	Tumor suppressor gene	Esophageal, colorectal, head, neck, laryngeal, and lung cancers	157, 158, 179, 245, 248, 249, 273	(71, 72)
PTEN	Tumor suppressor gene	Breast cancer (12%), thyroid cancer (1%), endometrial cancer (2.6%), renal cell cancer (1.6%), colon cancer (5%), and malignant melanoma (2%)		(73)
EGFR	Proto-oncogene	Glioblastoma, non-small-cell lung cancer, breast cancer, pediatric gliomas, medulloblastomas, and ovarian cancer	Exon 19 deletion, exon 21 point mutation, exon 18 point mutation, and exon 20 insertion	(74, 75)

### 4.1 KRAS

Approximately 30% of human tumors carry ras gene mutations, and the ras gene family includes Kras, Nras, and Hras. Kras is the most commonly mutated gene in the lung, colon, and pancreatic tumors (77), with a mutation rate of approximately 70%–90% in pancreatic cancer, 50% in colon cancer, 25%–50% in lung cancer (69), and 15%–25% in lung adenocarcinoma (78, 79). Point mutations (including G12D, G12V, G12C, G13D, AMP, and G12R) are the most common Kras mutations (70). Under normal circumstances, the activation and inactivation of Kras are finely regulated, as wild-type Kras is temporarily activated by tyrosine kinases such as active epidermal growth factor receptor (EGFR). Activated Kras can motivate downstream signaling networks to execute diverse bioactivities, and Kras is then rapidly inactivated (80). Mutant Kras proteins are uncontrollable, as they can be continuously activated in the absence of an EGFR activation signal, inducing uncontrolled cell proliferation and malignant invasion (80). Most mutations in lung cancer models are Kras dependent; Kras mutations are found in 90% of spontaneous and chemically induced lung tumors in mice, and genetically engineered Kras mice have been widely used in lung cancer research, which is very similar to the genetic and pathophysiological characteristics of human lung cancer (45, 81, 82). Johnson et al. constructed a new type of mouse with a potential KrasG12D allele (KrasLA), and mice carrying these mutations easily formed a variety of tumor types, predominantly lung tumors, and 100% of mice developed numerous kinds of lung tumors at an early stage (68). However, KrasLA mice were found to die of respiratory failure caused by lung lesions at a very young age, and the incidence of other tumors is difficult to predict, which restricts the application of this model (69). Kras gene mutations account for 6% and 18% of diffuse-type and intestinal-type gastric cancers, respectively (83). Brembeck et al. established Kras transgenic mice under the control of the cytokeratin 19 (K19) promoter; parietal cell decrease and mucous neck cell proliferation were found in 3- to 6-month-old mice (84). In pancreatic cancer, the Kras mutation occurs in the early stages of tumorigenesis and accounts for approximately 90% of pancreatic ductal adenocarcinomas; it is often combined with other classical mutations (PTEN, etc.) to induce pancreatic cancer, which will be mentioned in the following article (85).

### 4.2 TP53

Somatic mutations in the tumor protein P53 (TP53) gene are the most common changes in human cancers (86). The incidence of TP53 mutations in ovarian, esophageal, colorectal, head, neck, laryngeal, and lung cancers is 38%–50%, and the mutation rate is approximately 5% in primary leukemia, sarcoma, testicular cancer, malignant melanoma, and cervical cancer, and it is more common in advanced or invasive cancer subtypes (71). Most TP53 mutations are missense mutations, followed by truncated mutations, intra-frame mutations, and synonymous and uncoded mutations, which are mainly concentrated in known hot spots (the most common sites are 157, 158, 179, 245, 248, 249, and 273) (72). Normally,TP53 functions by activating or inhibiting the transcription of numerous critical genes in diverse bioactivities, including cell cycle arrest, DNA repair, metabolism, senescence, and apoptosis (86). Trp53 (TP53 is called Trp53 in mice) knockout mice are common transgenic animal models that formulate spontaneous tumors at the age of 6 months (including breast cancer, sarcoma, brain tumor, and adrenocortical carcinoma) (87). Compared with homozygous mice, mice with the Trp53 allele heterozygotes had later spontaneous tumors; the most common tumor type in homozygotes was malignant lymphoma, and the main heterozygotes were osteosarcoma and soft tissue sarcoma (88). In C57BL/6 or 129/Sv mice, the Trp53 deletion preferentially induces sarcoma and lymphoma, and the incidence of breast cancer has gradually increased from 21.4%-46.2% in the fourth generation after backcrossing with BALB/c mice for many generations (89). It has been reported that the incidence of gastric invasive adenocarcinoma in Trp53^−/−^ mice is significantly higher than that in WT mice (90). Ralph et al. established a mouse model of neuroendocrine lung tumors by conditionally inactivating Rb1 and Trp53 in mouse lung epithelial cells, and its morphology and immunophenotype were significantly similar to those of SCLC (91). However, increasing evidence shows that the TP53 gene still has antitumor effects after the loss of these classical activities (92). Using a mutant mouse model of p53-3KR (K120R, K161R, and K162R), researchers found that Trp53 can still inhibit cancer initiation, mainly by regulating cell metabolism, despite losing the antitumor effect of inducing cell cycle arrest, apoptosis, and senescence (93).

### 4.3 PTEN

Pten (phosphatase and tensin homolog deleted on chromosome ten) is a classical tumor suppressor gene with a mutation rate of approximately 12% in breast cancer, 1% in thyroid cancer, 2.6% in endometrial cancer, 1.6% in renal cell cancer, 5% in colon cancer, and 2% in malignant melanoma (73). The PTEN deletion was also reported in 15% of poorly differentiated serous ovarian cancers (94), and Pten mutations were also found in 20% of endometrioid ovarian cancers (95); approximately 53% of patients with primary bladder cancer showed a decrease or deletion of the Pten protein in the cytoplasm or nucleus of their tumor cells (96). The tumor inhibitory activity of PTEN depends to a large extent on its phosphatase activity, which regulates the activity of many important cellular pathways, such as PI3K/AKT; thus, it can regulate many cell processes, including proliferation, survival, energy metabolism, cell structure, and movement (97). The current transgenic model of Pten is widely used in the study of tumorigenesis mechanisms; however, half of the Pten knockout mice died within 1 year after birth, and the rest developed a variety of tumors, including lung, breast, thyroid, endometrial, and prostate cancer, and T-cell lymphoma (98). AlbCrePten (flox/flox) mice established by Horie et al. using the Cre-loxP system showed hepatocyte specific knockouts of Pten, and AlbCrePten (flox/flox) mice showed huge liver hypertrophy and steatohepatitis, as well as triglyceride accumulation similar to human nonalcoholic steatohepatitis (NASH); at 78 weeks of age, all AlbCrePten (flox/flox) mice had liver adenomas, and 66% of AlbCrePten (flox/flox) mice had HCC (99). Yanagi et al. specifically knocked out the Pten gene in bronchiolar alveolar epithelial cells of mice under the control of doxycycline to establish SOPten (flox/flox) mice, of which 90% of the SOPten (flox/flox) offspring mice died of hypoxia shortly after birth (100). Ninety weeks later, all SOPten (flox/flox) mice born showed significant visible lung tumors: 13 tumors were adenocarcinoma and 1 tumor was SCC, as determined by histological examination (100). Russo et al. found that the Pten deletion increases cell migration, invasion, and upregulation of WNT4, which is a key regulator of Müllerian duct development during embryogenesis (101). Tsuruta et al. used the Cre-loxP system to specifically knock out the Pten gene in the urine epithelium of mice to obtain FPten (flox/flox) mice; histologically, the urine epithelium cells of the mice showed enlargement of the nucleus and cell volume, and ultimately, approximately 10% of FPten (flox/flox) mice spontaneously developed into pedicled papillary transitional cell carcinoma (TCC) (96).

### 4.4 EGFR

Epidermal growth factor receptor (EGFR) is a member of the HER family, which includes HER1 (erbB1, EGFR), HER2 (erbB2, NEU), HER3 (erbB3), and HER4 (erbB4) (102). Studies have shown that there is high or abnormal expression of EGFR in many solid tumors, which is related to tumor cell proliferation, angiogenesis, invasion, metastasis, and inhibition of apoptosis (103). In a study using EGFR knockout mice, the types of cells most affected by the EGFR deletion were epithelial cells and glial cells, while the types of cells overexpressing EGFR in the human tumors were epithelial cells and glial cells (104, 105). A large number of EGFR mRNA deletions have been observed in a number of neoplasms, first in glioblastoma, but recently in non-small-cell lung cancer, breast cancer, pediatric gliomas, medulloblastomas, and ovarian cancer (74). There are four main types of EGFR mutations: exon 19 deletion, exon 21 point mutation, exon 18 point mutation, and exon 20 insertion (75). The most common EGFR mutations are the exon 19 deletion mutations (19DEL) and exon 21 mutations (21L858R), followed by exon 18 G719X, exon 20 S768I, exon 21 L861Q mutation, and T790M mutation in exon 20, which are associated with acquired drug resistance in the first and second generations of EGFR-TKIs (75). EGFR knockout mice were stunted and died at different developmental stages (implantation, second trimester, or early postpartum), and mainly characterized by epithelial cell defects (including skin, lung, gastrointestinal tract, teeth, and eyelid defects), impaired intestinal proliferation, reduced stem cell area, and mucosal structure disorder (105). In order to study the role of activated EGFR mutations in lung cancer, Ohashi et al. established transgenic mice carrying EGFR mutations (five nucleotides encoding five amino acids were deleted, which was equivalent to the EGFRdelEN746-A750 mutation found in lung cancer patients) specifically in type II alveolar epithelial cells through its specific SP-C promoter and found that 9 of 47 newborn mice were positive for EGFR gene mutations, 3 mice of the positive type developed lung adenocarcinoma, only 1 mouse carrying lung adenocarcinoma could reproduce, and all of its offspring developed lung tumors after 7 weeks (106). Ohashi et al. also used EGFR-mutated mouse models to study the evolution of lung adenocarcinoma. Transgenic mice were killed at different time points for pathological examination; atypical adenomatoid hyperplasia (AAH) appeared at 3–4 weeks, diffuse bronchiolo-alveolar carcinoma (BAC) appeared at 4–5 weeks, adenocarcinoma with solid features was observed at 7 weeks, and multiple tumor nodules were observed on the lung surface (106). Ohashi et al. used a similar method to construct transgenic mice expressing EGFRL858R in type II alveolar epithelial cells; 8 mice had L858R deletion mutations in 27 newborn mice, and 2 mice with the positive mutation developed multifocal adenocarcinomas at 7 weeks. All the offspring of these two mice had BAC at 4–5 weeks and adenocarcinomas at 7 weeks (107).

### 4.5 Combined mutations

Tumors are often caused by multiple gene mutations, the most common of which is the activation of oncogenes and inactivation of tumor suppressor genes (108). Kras and TP53 are common combined gene mutations in human cancer, and KP mice with both Kras and Trp53 mutations are the most classic transgenic model; all KP mice developed primary lung tumors. Six weeks after the lung lesions of mice developed from atypical adenomatous hyperplasia to lung adenomas, the tumors of these mice showed a high degree of nuclear atypia, causing interstitial connective tissue hyperplasia, invasiveness, and metastasis (109). Kras mutations were found in 95% of pancreatic ductal adenocarcinomas. KPC mice were triple mutants with aKras^LSL-G12D^, p53^LoxP^, and Pdx1-CreER for tamoxifen-inducible pancreatic ductal adenocarcinoma. Pancreatic ductal metaplasia and pancreatic intraepithelial neoplasia occurred in KPC mice at 8–10 weeks, and invasive pancreatic ductal adenocarcinoma occurred at 14–16 weeks, and metastasized to the liver, lung, and peritoneum (110). Combined mutations of Kras and Pten are also common in human cancers, and the Ptf1a^Cre-ERTM^, Kras^LSL-G12D^, and Pten^flox^, tamoxifen-inducible triple mutant strain (KPP) may be useful as a model for pancreatic adenocarcinoma (PDA)-induced cachexia-a wasting syndrome characterized by the pronounced loss of skeletal and cardiac muscle and adipose tissues (111). Mutation activation of BRAF is the earliest and most common genetic change in human melanoma; mice specifically expressing BRAF(V600E) showed benign melanocyte proliferation, but did not develop melanoma after 15–20 months, and BRAF(V600E) expression combined with PTEN gene silencing could induce malignant melanoma and metastasis to lymph nodes and lungs (112). The Rb1 deletion, TP53 deletion, and Myc amplification are all common mutations in SCLC (113). RPM mice carried three gene mutations: Rb1^fl/fl^, Trp53^fl/fl^, and Myc^LSL/LSL^; 100% of these mice developed SCLC after 6–8 weeks (113). It is worth noting that these common classical gene mutations are often combined with new gene mutations to study the function of these new genes in carcinogenesis.

## 5 Transplantable animal tumor models

**Figure 3:**
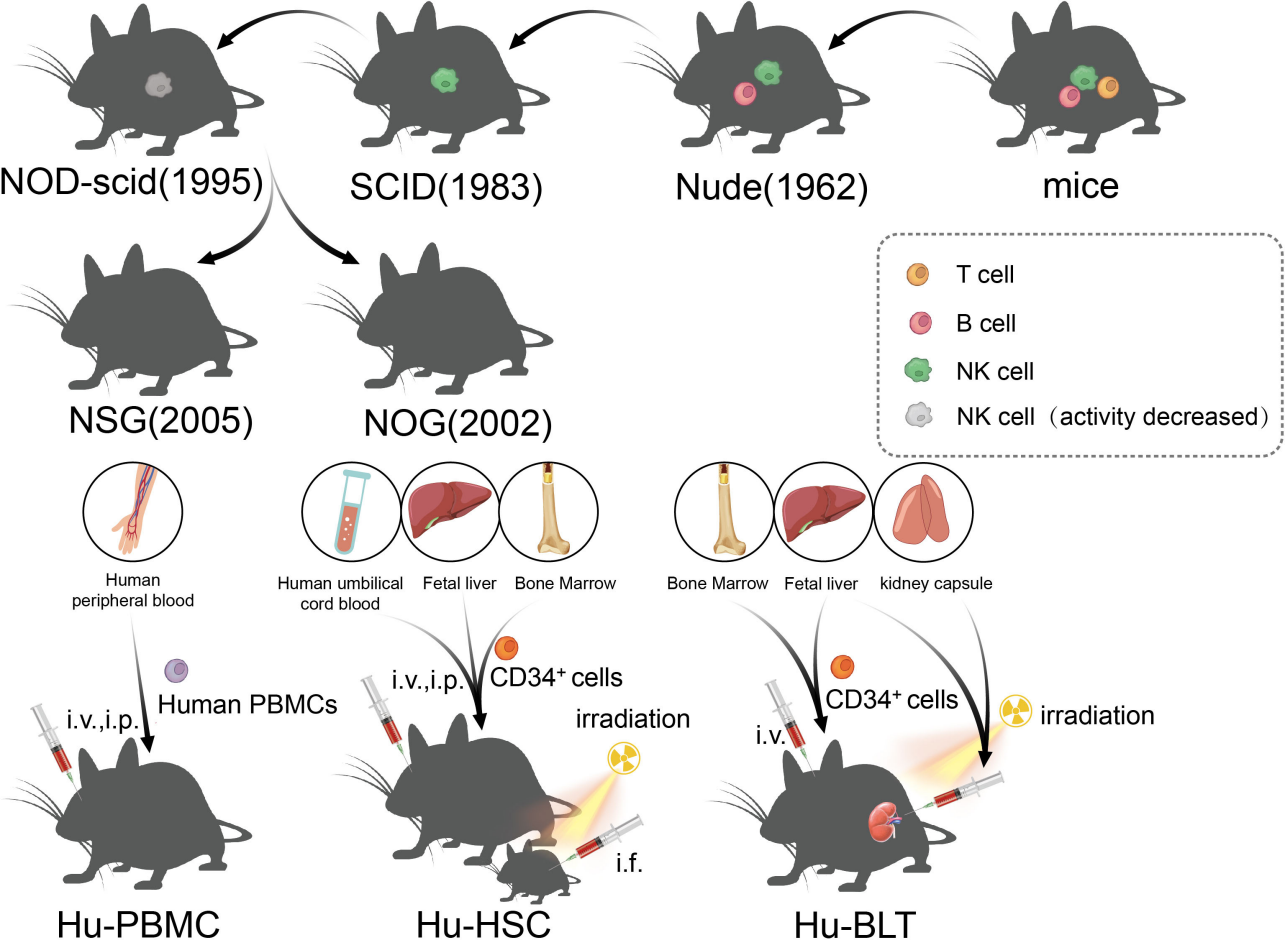
The development of immunodeficient mice and the construction method of the humanized mice model.

Human tumor generated mouse models are established by transplanting human tumor cells and/or tissues of research interest into recipient animals (almost always possessing immune function deficiencies) (**Figure 3**). The advantage is that most types of human tumors can establish transplantable tumor models in immunodeficient animals, and under the same inoculation conditions, the growth rate of animals is the same, the difference in tumor formation rate is small, and the inoculation tumor formation rate is high. The disadvantage is that the recipient host animal needs to be in an immunodeficient state requiring special housing in an aseptic environment, which is expensive to maintain. Furthermore, not all cell types of human tumors can be successfully established in rodent models and the stroma of the human tumor tissue obtained may contain the components of the recipient animals. It is worth noting that the key to developing an optimal transplanted tumor model lies in the immune status of the host (immunodeficiency state) and the composition of the graft (containing important or all components of the tumor).

### 5.1 Immunodeficient mice

The immune status of the host has undergone many improvements, which is the key to the development of a successful transplant tumor model. Transplantable tumors are divided into allogeneic transplantation and xenogeneic transplantation, and the most used is the xenotransplantation of human tumors. Due to the existence of immune rejection, wild mice cannot be used for xenotransplantation of human tumor. However, human tumors can be implanted in immunodeficient mice, and the tumors can maintain the histological, immunological, and biological characteristics of original tumors. Nude mice were first identified by Grist in 1962, and they were called nude mice as they are hairless. Flanagan found that nude mice lack thymus and T lymphocytes, and thus, they lacked T cell-mediated immune responses and could be used as recipients for human tumor xenotransplantation (114). However, because nude mice still have B cells and NK, they are not suitable for use as hosts for lymphoma and leukemia, which limits the extensive application of nude mice in relation to transplanted tumors. Severe combined immunodeficiency (SCID) mice were first reported by Bosma in 1983 (115). SCID mice are more severely immunodeficient than nude mice, and the mutant genes of the SCID mice were identified in 1996. The maturation defects of B lymphocytes and T lymphocytes in SCID mice were caused by a point mutation in the Prkdc (protein kinase, DNA-activated, catalytic subunit) gene, which causes the affected lymphocytes to die prior to maturation or be cleared by the macrophages, granulocytes, and NK cells in the body (116, 117). However, it is usually difficult to establish a successful model for lymphoma and metastatic tumors in SCID mice because of leakage (some SCID mice will restore the function of some T and B lymphocytes with age) (118). In addition, NK cells and other innate immunoreactive substances in SCID mice were present at high levels, which limits the success rate of transplantation, such as human hematopoietic stem cell (HSC) transplantation (119). Subsequently, the establishment of NOD/SCID mice ushered in a new breakthrough in transplanted animal tumors, which are a type of spontaneous type 1 diabetic mice, caused by T lymphocytes infiltrating and destroying islets, as well as complement loss and impaired function of NK, macrophages, and dendritic cells (120, 121). NOD/SCID mice were established through the hybridization of NOD and SCID mice, and NOD/SCID mice will not develop type 1 diabetes, as they lack an adaptive immune system and the loss of effector T cells; moreover, due to extensive defects in innate and adaptive immunity (low activity of NK cells and loss of T- and B-cell function), NOD/SCID mice have become a stable and excellent animal model for human HSCs and human solid tumor transplantation (122). At the beginning of the 21st century, scientists introduced IL-2Ry mutations on the basis of NOD/SCID mice, resulting in NSG and NOG mice, which, in addition to being the mice with the highest degree of immunodeficiency at present, could also be used to construct humanized immune system mice to study the antitumor effects of chimeric antigen receptor T-cell immunotherapy (CAR-T) and immune checkpoint inhibitors (ICIs) (123), and will be discussed in detail later.

### 5.2 PDX

Transplant components have also been the focus of transplantable tumor model research in recent years, and in the past, we transplanted tumor cell lines into animals, which are simpler and easier to use, but the defects cannot be ignored: first, this tumor model cannot fully represent the unique characteristics of each cancer patient; furthermore, this model cannot reconstruct the remaining non-tumor cell components in the tumor tissues, which plays an important role in tumorigenesis and development. Consequently, the patient-derived xenotransplantation (PDX) model has been actively generated and applied, and the PDX model can directly implant cancerous tissues from the patient’s tumor into immunodeficient mice, thus preserving both the cell–cell interaction and the tumor microenvironment (124). The PDX model retains the characteristics of the primary patient tumor, including the gene expression profile and drug response, and offers great advantages for drug screening, biomarker development, and the evaluation of therapeutic effects (125). The PDX model usually takes approximately 6 months to 2 years to establish, and the success rate varies (10%–90%), depending on the tumor source and disease characteristics (125). Specifically, invasive, recurrent, and the transplantation rates tend to be higher for highly metastatic tumors (126). Gastrointestinal tumors (such as colon and pancreatic cancers) have higher transplantation success rates than other cancers, and the transplantation rate in breast cancer is low (127). The success rates of the PDX model is as follows: colon cancer [63.5% (54/85) in nude mice and 87% (74/85) in NOD/SCID mice] (128, 129), pancreatic cancer [61% (42/69) in nude mice and 67% (8/12) in SCID mice] (130, 131), and breast cancer [12.5% (25/200) in nude mice and 27% (13/49) in NOD/SCID mice] (132, 133).

Animal models also play an important role in drug development and screening. In the past, the average success rate of translating animal research into human clinical trials was approximately 8%, and <5% of antineoplastic drugs were approved to enter the market (134, 135). Although the accuracy of the PDX model on drug efficacy and drug resistance was up to 90% (136), the traditional PDX model takes a long time to construct; owing to this, it cannot quickly reflect the drug sensitivity of patients and cannot meet clinical needs. China Lidi Biological has developed a new rapid PDX drug sensitivity detection technique (OncoVee ^®^MiniPDX) for screening clinically related programs of cancer, which uses patient-derived tumor cells arranged in hollow fiber capsules after 7 days of subcutaneous culture. The active morphology and pharmacokinetics of the tumor cells in MiniPDX capsules were evaluated systematically, and the morphological and histopathological characteristics of the tumor cells in MiniPDX capsules were consistent with those of the PDX model and primary tumor (137).

### 5.3 Humanized mice model

Recently, ICI and CAR-T have enabled new breakthroughs in the treatment of tumors (138, 139). Although PDX, which depends on immunodeficient mice, has been widely used in the research of tumor immunity and the development of new therapies, the lack of models for the human immune system and tumor immune microenvironment limits the study of immune mechanisms and transformation of immunotherapy to a great extent. The humanized mouse model is a mouse model that reconstructs the human immune system by implanting human hematopoietic cells, lymphocytes, or tissues into immunodeficient mice, which can effectively reconstruct the human immune system and better simulate the characteristics of human immunity (140). From the earliest nude mice to the later SCID and NOD/SCID mice, the success rate of human cell implantation has been low either due to the existence of innate immunity or because of high sensitivity to radiation and the limited life cycle. Owing to this, all these models limited the application of immune system-humanized mice in practical research to some extent. In NOG mice, mature T/B cells and NK cells are lacking, complement activity is decreased, and the function of macrophages and dendritic cells is impaired; therefore, NOG mice are an ideal model for human immune cell transplantation (141). Subsequently, the IL-2 receptor γ mutation was further developed. NSG mice had a complete IL-2 receptor γ chain-invalid allele, similar to NOG mice, with a loss of T/B and NK cells, lack of complement activity, and defects in macrophages and dendritic cells (142). In addition, other highly immunodeficient mice, such as BRG and BRGS mice, have been established as humanized mouse models that lack T/B cells and NK cells (143). Although NOG and NSG mice are very useful humanized models, they still lack the ability to reconstruct myeloid and NK cells, and a new generation of super immunodeficiency models HuNOG-EXL and HuNSG-SGM3 have been developed, which can express some growth factors to promote myeloid regeneration (142, 144). According to the method of human immune system reconstruction, the humanized mouse model can be divided into three categories: Hu-PBMCs (humanized-peripheral blood mononuclear cells), Hu-HSCs (humanized-hematopoietic stem cells), and Hu-BLTs (humanized-bone marrow, liver, thymus) (145). The Hu-PBMC model is a simple and economical humanized mouse model in which mature lymphocytes from peripheral blood mononuclear cells (PBMCs) are injected into immunodeficient host mice through intraperitoneal (i.p.) or intravenous (i.v.) injection (145). This method was first described in CB17-SCID mice in 1988 and has been widely used to study the human immune response in autoimmunity and infectious diseases (117). However, graft-versus-host disease (GvHD) occurs in the Hu-PBMC model in 2–3 weeks, and the survival time is short (146). At present, this model is often used to study the activation of human effector T cells and to evaluate immunosuppressive drugs; in the Hu-HSC mouse model, human CD34^+^ HSCs from human umbilical cord blood, adult bone marrow, or fetal liver were injected into adult mice (intravenous or intrafemoral) or newborn mice (intracardiac or intrahepatic) (147, 148), and sublethal irradiation of host mice is needed to eliminate HSC and promote the transfer of human HSC (145). Fetal liver and umbilical cord blood are the most commonly used sources of human CD34^+^ HSCs, which are easier to colonize in immunodeficient mice than that in adult HSCs. Although this method can produce a variety of HSCs in adult mice, the number of T cells produced is small and the model does not possess functional immune cells (149). In newborn mice (less than 4 weeks old), good human cell transplantation can be obtained, and T cells, B cells, macrophages, NK cells, and DCs can be produced (150). At present, it is believed that the Hu-HSC model can establish the human innate immune system and lymphocytes, with little or no occurrence of GvHD, which can be used for long-term research; however, owing to the species differences between humans and mice, there is a lack of human cytokines in mice, and the development of human stem cells in mice is limited (149). The method of establishing Hu-BLT is to transplant human fetal liver and thymus tissue into the renal capsule of adult immunodeficient recipient mice after sublethal irradiation, and at the same time, fetal liver or bone marrow-derived CD34^+^ HSCs from the same individual is injected intravenously (i.v.) into recipient mice (151). The Hu-BLT model is often used to study adaptive immune responses, such as HIV infection. However, the incidence of GvHD in the Hu-BLT model is higher than that in other CD34^+^ HSC transplantation models (145), and complex and precise surgical procedures are required, thus limiting the application of the Hu-BLT model in the research and development of tumor immune drugs.

## 6 Prospects

The use of animal models to develop human healthcare can be traced back to the 6th century BC (152). During this time, there have been significant developments in biotechnology and animal models that have contributed to the development of mechanisms of disease and drug discovery. Since the establishment of the earliest spontaneous animal tumor model, we have obtained a great deal of information about tumor generation and progression. However, we have gradually eliminated this method because of its randomness, high cost, and long cycle of tumorigenesis. Then, we used chemical drugs to induce tumors in mice to establish tumor models and study the carcinogenic effect of a certain factor. In fact, induced tumor is the most widely used method for establishing animal tumor models at present, due to the low technical requirements and cost, most of the laboratories can perform this biotechnique. With the development of the transgenic animal model and the PDX model, we have made great progress in understanding specific gene function, building animal models of human diseases, and evaluating the safety and effectiveness of new drugs, and it is hopeful that it will be a bright light for the study of animal tumor models in the future.

However, they still have significant limitations in modeling human cancer, which is mainly reflected in the immune microenvironment and tumor microenvironment, or the more subtle differences caused by species-specific differences. Although we have developed the PDX model and humanized model to reduce these differences, it is urgent that we develop and establish more effective animal tumor models to facilitate more detailed investigation of the tumor development process.

## Author contributions

All authors contributed to the article and approved the submitted version.
